# Angiogenic Potential of Human Neonatal Foreskin Stromal Cells in the Chick Embryo Chorioallantoic Membrane Model

**DOI:** 10.1155/2015/257019

**Published:** 2015-06-29

**Authors:** Radhakrishnan Vishnubalaji, Muhammad Atteya, May Al-Nbaheen, Richard O. C. Oreffo, Abdullah Aldahmash, Nehad M. Alajez

**Affiliations:** ^1^Stem Cell Unit, Department of Anatomy, College of Medicine, King Saud University, Riyadh 11461, Saudi Arabia; ^2^Histology Department, Faculty of Medicine, Cairo University, Cairo, Egypt; ^3^Saudi Electronic University, Saudi Arabia; ^4^Bone and Joint Research Group, Centre for Human Development Stem Cells and Regeneration, Human Development and Health, Institute of Developmental Science, University of Southampton, Southampton, UK; ^5^KMEB, Department of Endocrinology, University of Southern Denmark, Odense, Denmark

## Abstract

Several studies have demonstrated the multipotentiality of human neonatal foreskin stromal cells (hNSSCs) as being able to differentiate into adipocytes and osteoblasts and potentially other cell types. Recently, we demonstrated that hNSSCs play a role during* in vitro* angiogenesis and appear to possess a capacity to differentiate into endothelial-like cells; however, their angiogenic potential within an *ex vivo* environment remains unclear. Current study shows hNSSCs to display significant migration potential in the undifferentiated state and high responsiveness in the* in vitro* wound healing scratch assay. When hNSSCs were seeded onto the top of the CAM, human von Willebrand factor (hVWF), CD31, smooth muscle actin (SMA), and factor XIIIa positive cells were observed in the chick endothelium. CAMs transplanted with endothelial-differentiated hNSSCs displayed a higher number of blood vessels containing hNSSCs compared to CAMs transplanted with undifferentiated hNSSCs. Interestingly, undifferentiated hNSSCs showed a propensity to differentiate towards ectoderm with indication of epidermal formation with cells positive for CD1a, CK5/6, CK19, FXIIIa, and S-100 cells, which warrant further investigation. Our findings imply a potential angiogenic role for hNSSCs *ex vivo* in the differentiated and undifferentiated state, with potential contribution to blood vessel formation and potential application in tissue regeneration and vascularization.

## 1. Introduction

Angiogenesis is a multifaceted process that involves endothelial cell proliferation, migration and differentiation, extracellular matrix (ECM) remodelling, and the functional development of new blood vessels from preexisting vasculature. The exploration of angiogenesis offers new approaches to understanding the mechanisms underlying vascular disease and to aid in regeneration. Furthermore, stem cell transplantation has emerged in the last few years as a potential therapy for several diseases, given the potential of stem cells to differentiate into multiple lineages and the prospect that they may offer trophic support for cell survival, tissue restoration, and functional improvement [1–3].

Mesenchymal stem cells or multipotent stromal cells (MSCs) are nonhematopoietic stem cells with extensive self-renewal and multilineage differentiation potential [4–7]. In our previous study, hNSSCs were shown to express thirty-three CD markers including known stromal cell-associated as well as several novel markers [6]. Moreover, these cells could be induced to differentiate into cells expressing endothelial markers and to form densely packed large diameter tubules during* in vitro* angiogenesis assay [5, 8]. However, the angiogenic capacity of hNSSCs* ex vivo* remains unclear.

Autologous stem cell transplantation has been employed to aid therapeutic angiogenesis in various diseases, including ischemic cardiac and limb disease and connective tissue disorders. Nonetheless, there is substantial heterogeneity in the system of recruitment, collection, and storage of autologous clinical grade source [9]. Our preliminary studies using neonatal foreskin showed promising results indicating that hNSSCs could be an alternative potential source for cell based angiogenesis [6, 8]. Thus, improved understanding of the cellular mechanisms of hNSSCs vasculogenesis and angiogenesis could offer new therapeutic approaches for hNSSCs.

The current study has examined the angiogenic potential of hNSSCs in an* ex vivo* angiogenic assay. The chick chorioallantoic membrane (CAM) assay offers excellent nutrient supply given the dense capillary network and preexisting vasculature providing a robust angiogenic* ex vivo* model to assay cells, scaffolds, and growth factors including a foundation of vessels that expand into implanted hNSSCs [10–13]. The assay is robust and economical, and, critically, the chick immune system is not fully developed allowing analysis of cells and materials without issues of immune rejection. Furthermore, the model has been used to investigate the efficiency and mechanisms of action of pro- and antiangiogenic natural and synthetic materials [10, 14, 15]. Thus we have used the CAM model to investigate the functional potential of hNSSCs to contribute to angiogenesis in an* ex vivo* environment.

## 2. Methodology

### 2.1. Ethics Statement

The use of human specimens in current study was approved by the Institutional Review Board at King Saud University College of Medicine (10-2815-IRB). The embryonic chicken chorioallantoic membrane assay was carried out at the University of Southampton according to Home Office Approval UK under the Project license—PPL 30/2762.

### 2.2. Isolation and Culture of hNSSCs

**Figure 1:**
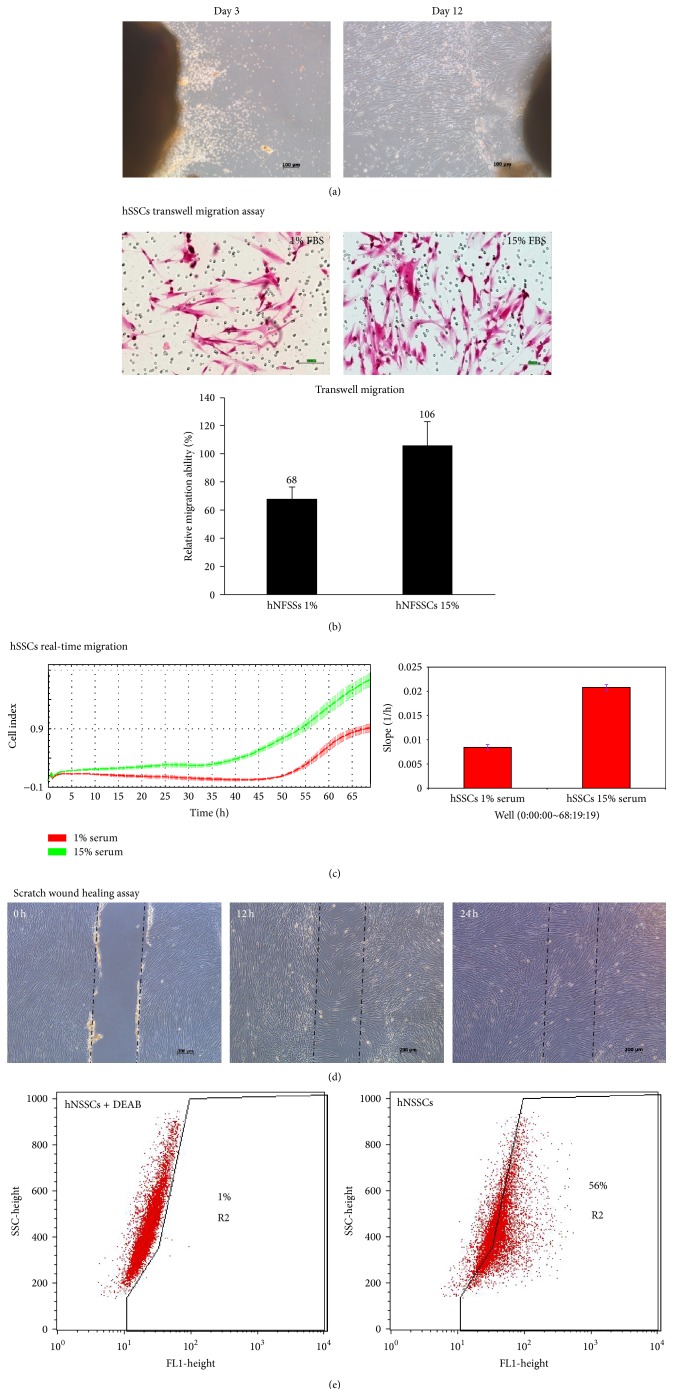
Explant organ culture system of neonatal foreskin. (a) Cell migration from skin tissue after 3 days (left); fibroblast-like cells can be observed migrating and sprouting out from tissue after 12 days (right). (b) A transwell migration assay where cells migrated to the lower chamber visualized by Eosin staining. The transwell migration assay was repeated and the total number of cells that migrated was quantified to determine the relative numbers of migrated cells (b, lower panel). Data are presented as mean ± S.D. The experiment was run in triplicate. (c) The real time migration was executed using the xCELLigence RTCA DP device. Cells were seeded per well in 16-well microelectronic sensing, two-chamber transwell plates (CIM-plates). The electrical impedance was captured every 15 min for an experimental duration of ~68 h. The rate of migration is expressed as the CI or the change in electrical impedance at each time-point (c, left). Values are expressed as the ±SEM of the 8 replica wells from three independent experiments (c, right). (d) Measurement of undifferentiated hNSSCs cell migration by* in vitro* wound healing scratch assay. The migration ability was assessed 12 h and 24 h from injury. The figure shows the migration of undifferentiated cells immediately after the scratch 0 h, 12 h, and 24 h (Bar = 100 *μ*m). (e) ALDH activity of hNSSCs was measured using the Aldefluor assay and FACS analysis. Cells incubated with specific inhibitor of ALDH, DEAB, were used to establish the baseline fluorescence of these cells and to define the Aldefluor positive region.

hNSSCs were isolated and cultured in accordance with our previously published protocols [6, 8]. In brief, cells were isolated by explant organ culture to establish outgrowth cell culture (Figure 1(a)). Newborn foreskins were received from voluntary circumcisions with informed consent. Tissues were washed and the epidermis was removed followed by the dermis. Tissues were placed in culture dishes with the epidermis layer facing upwards and the dermis area in contact with the plastic surface with a droplet of culture medium. Cultures were maintained at 37°C and 5% CO_2_ in a humidified environment. Additional media were added following cell attachment and culture was maintained for 7 days or until outgrowths of fibroblast-like spindle shaped cells were observed. At 70–80% confluency, cells were trypsinized and residual tissues were removed. The culture medium consisted of Dulbecco's Modified Eagle Medium (DMEM) supplemented with D-glucose 4500 mg/L, 4 mM L-Glutamine, and 110 mg/L Sodium Pyruvate, 10% Fetal Bovine Serum (FBS), 1x penicillin-streptomycin (Pen-strep), and nonessential amino acids (all purchased from Gibco-Invitrogen, USA).

### 2.3. Aldefluor Assay

The Aldefluor kit (Stem Cell Technologies, Vancouver, BC, Canada) was used to determine the percentage of cells with high Aldehyde Dehydrogenase (ALDH) enzymatic activity. Briefly, 10^6^ cells were resuspended in Aldefluor assay buffer containing ALDH substrate as recommended by the manufacturer. As a negative control for all samples, an aliquot of “Aldefluor-exposed” cells was immediately quenched using an ALDH inhibitor, diethylaminobenzaldehyde (DEAB). After 30 minutes of incubation at 37°C, the cells were centrifuged and resuspended in 500 *μ*L Aldefluor buffer and analyzed using BD FACS Calibur flow cytometer (BD Biosciences). Aldefluor staining was detected within the green fluorescence channel FL1. Samples treated with the inhibitor DEAB (+DEAB) were used as controls to establish the gates defining the ALDH^+^ region. Cell Quest Pro Software Version 3.3 (BD Biosciences) was used to analyze the data.

### 2.4. Transwell Migration Assay

The hNSSCs transwell migration assay was performed as we described before [16]. Briefly, transwell membrane (8 um pore; BD Falcon) inserts were used in the study. The undifferentiated cells were trypsinized, washed, and resuspended in medium without FBS. To the lower wells of the chambers, migration-inducing medium (with 1% and 15% FBS) was added. The upper wells were filled with serum-free medium containing cells (1 × 10^5^ cells per well). The chamber was incubated at 37°C and 5% CO_2_ in a humidified environment. After 24 h, assays were stopped with removal of the medium from the upper wells and careful removal of the filters. Filters were fixed using 4% paraformaldehyde and stained with eosin. Assessment of completed transmigration was performed by microscopy, and random fields were examined (four fields per filter) for the presence of cells on the lower membrane side only (Carl Zeiss-Axio Observer.1 equipped with a digital camera (Axiocam MRc5)). The number of migrated cells was counted using Image-Pro Plus software (Media Cybernetics).

### 2.5. The hNSSCs Real-Time Migration

Real-time analysis of cell migration was executed using the xCELLigence RTCA DP system (ACEA Biosciences, San Diego, CA). 40,000 cells were seeded per well in a 16-well microelectronic sensing, two-chamber transwell plates (CIM-plates) containing the respective serum conditions. Medium containing 15% serum (chemoattractant) and 1% serum (control) was added to the bottom wells. Migration of cells is measured as a result of the interaction of cells with the electrodes on the bottom surface of top chamber and represented as a change in cell index (CI), an arbitrary unit derived from the relative change in electrical impedance across microelectronic sensor arrays. The electrical impedance was captured every 15 min for an experimental duration of ~68 h. The rate of migration is expressed as the CI or the change in electrical impedance at each time-point. Values are expressed as the ±SEM of the 8 replica wells from three independent experiments.

### 2.6. *In Vitro* Scratch Assay (Wound Healing Model)

For the assessment of cell migration, confluent undifferentiated hNSSCs maintained in standard medium were wounded with a plastic micropipette tip (yellow tip; 20–200 *μ*L). After washing, the medium was replaced by fresh medium. Photographs of the wounded area were taken after 12 and 24 hrs using phase-contrast microscopy. For evaluation of wound closure, four randomly selected points along each wound area were marked, and the horizontal distance of migrating cells from the initial wound was measured (Carl Zeiss-Axio Observer.1 equipped with a digital camera (Axiocam MRc5)).

### 2.7. Endothelial Differentiation

hNSSCs were differentiated as previously detailed [5]. At 70–90% confluency medium was replaced with endothelial induction medium (Medium 199 with Earle's salt with L-Glutamine (PAA; Cat. Number E15-834) + 10% Fetal Calf Serum (FCS), 1% Pen-strep, 2 ng/mL VEGF (R&D systems, USA), and ECGS/H 3 mg/mL protein with 22.5 mg/mL Heparin (endothelial cell growth supplements (ECGS), Promocell, UK) for 7 days). The medium was changed every 2 days.

### 2.8. Organotypic Culture

hNSSCs were cultured on the CAM as previously detailed allowing the analysis of hNSSCs in an* ex vivo* culture (Figure 3) [12]. After Trypsinization, cell pellets were resuspended in 10 mL culture medium (control cells in routine culture medium, induced cells in endothelial induction medium). Cell count was performed to give a cell density of between 3 × 10^5^ and 5 × 10^5^/mL. One mL of cell suspension was added per falcon tube, centrifuged at 400 g for 10 minutes and incubated at 37°C, supplemented with 5% CO_2_. After 2 days, pellets formed were cultured for 2 more days on organotypic culture until cell attached to the confetti (Figures 3(a) and 3(b)) (hydrophilic PTFE (polytetrafluoroethylene) membranes (0.4 mm pore size); Millipore; UK).

### 2.9. *Ex Vivo* Angiogenesis Using the Chick Chorioallantoic Membrane Assay

The chick chorioallantoic membrane (CAM) was exposed by cutting a window (2 cm^2^) on one side of 10-day-old specific pathogen-free chicken egg (Figure 3(c)). Confetti organotypic culture was placed on the CAM. The window in the shell was sealed with adhesive tape and the egg incubated for 10 days. Representative CAMs from each group (untreated and treated) were imaged using a dissecting microscope (10x) and counted (Figures 3(d), 3(e), 3(f), and 3(g)). The number of fine distinct blood vessel branch points in the area of the confetti was counted [17]. As angiogenesis is categorized by the sprouting of new vessels from preexisting vessels in response to hNSSCs, thus counting blood vessel branching points is a functional quantitative means of determining angiogenic index. At least 5 embryos were used per group. Data were assessed in terms of average number of branching points per group ± standard deviation.

### 2.10. Histological and Immunohistochemical Analysis of CAM

Representative CAMs from each group were excised and fixed in formalin and subsequently dehydrated through a series of graded alcohols and embedded in low-melting point paraffin using an automated Shandon Citadel 2000. Tissue sections 6 *μ*m thick were cut from across the CAM contacting confetti and stained for the light green and alcian blue counter stain, followed by staining with CD 31 (CD 31; 1 : 50; Proteintech Europe) and vWF (1 : 200; Dako) to determine angiogenesis. For further analysis, formalin-fixed paraffin-embedded CAM were stained according to the manufacturer's staining protocol on a Bondmax fully automated IHC and ISH staining system (Leica Microsystems GmbH, Germany) and stained with haematoxylin and eosin (H&E). Antibodies were from Novocastra (Leica Biosystems) ready to use except CD1a (Clone MTB1; PA0235), CK19 (Clone b170; PA0799), FXIIIa (Clone E980.1; PA0449), S-100 (Polyclonal; PA0900), SMA (alpha sm-1; PA0943), and CK5/6 (Clone D5 & 16B4; 356 M) which were from Cell Marque and were used according to manufacturer's standard protocols. Slides were digitized using high-resolution whole-slide digital ScanScope scanner (Aperio Technologies, Inc.). Mouse monoclonal (EMR8-5; ab70328; Abcam) to HLA Class 1 ABC was used to identify the human cells in CAM. All the staining was performed with respective positive control tissues according to the antibody manufacturer instructions and without primary antibodies; the secondary antibody staining was considered as experimental negative control (Supplementary Figures 1 and 2 in Supplementary Material available online at http://dx.doi.org/10.1155/2015/257019). The digital slide images were viewed and analyzed using Aperio's ImageScope software (Aperio Technologies, Vista, CA, USA).

### 2.11. Statistical Analysis

All measurements were calculated as mean percentage ± standard deviation. Statistical analysis was performed using Microsoft Excel 2007 software. Differences among groups were determined using *t*-test and statistical differences were considered to be significant if *P* ≤ 0.05. All experiments were performed a minimum of three times.

## 3. Results

### 3.1. hNSSCs Possess High Migration Potential* In Vitro* and Are Enriched in ALDH^+^ Cells

The outgrowth of fibroblast-like spindle shaped cells was observed in neonatal foreskin explants organ cultures as early as three days after culture with extensive cell outgrowth observed by day 12 (Figure 1(a)). We previously characterized the phenotype, colony forming unit, population doubling, and differentiation potential of hNSSCs in comparison with conventional bone marrow-derived MSCs [6, 8]. We subsequently examined the migration potential of hNSSCs using a manual transwell and automated real-time migration system. hNSSCs were seeded in the upper chamber of a transwell migration system (8.0 *μ*m pore size) in the manual assay and we followed the manufacture protocol in automated system, and media (DMEM) supplemented with 15% FBS were used as an attractant. As illustrated in Figures 1(b) and 1(c), naive hNSSCs displayed high migration potential* in vitro*. Similarly, hNSSCs also exhibited enhanced migration potential when assessed using an* in vitro* wound healing assay at both 12 and 24 hrs after scratch (Figure 1(d)). The presence of potential stem cells within hNSSC cultures was assessed using the Aldefluor assay, which has been utilized to examine the frequency of stem cells in a number of cell types which typically display high ALDH activity [18, 19]. The ALDH inhibitor (DEAB) was used to ensure specificity of the assay and appropriate FACS analysis. FACS analysis indicated the frequency of ALDH+ cells in hNSSCs to be around 52 ± 0.9% (Figure 1(e)). These results suggest high frequency of undifferentiated progenitor/stem cells within the hNSSC cultures.

### 3.2. Differentiated and Undifferentiated hNSSCs Displayed Different Morphological Changes When Cultured on Different Surfaces

**Figure 2:**
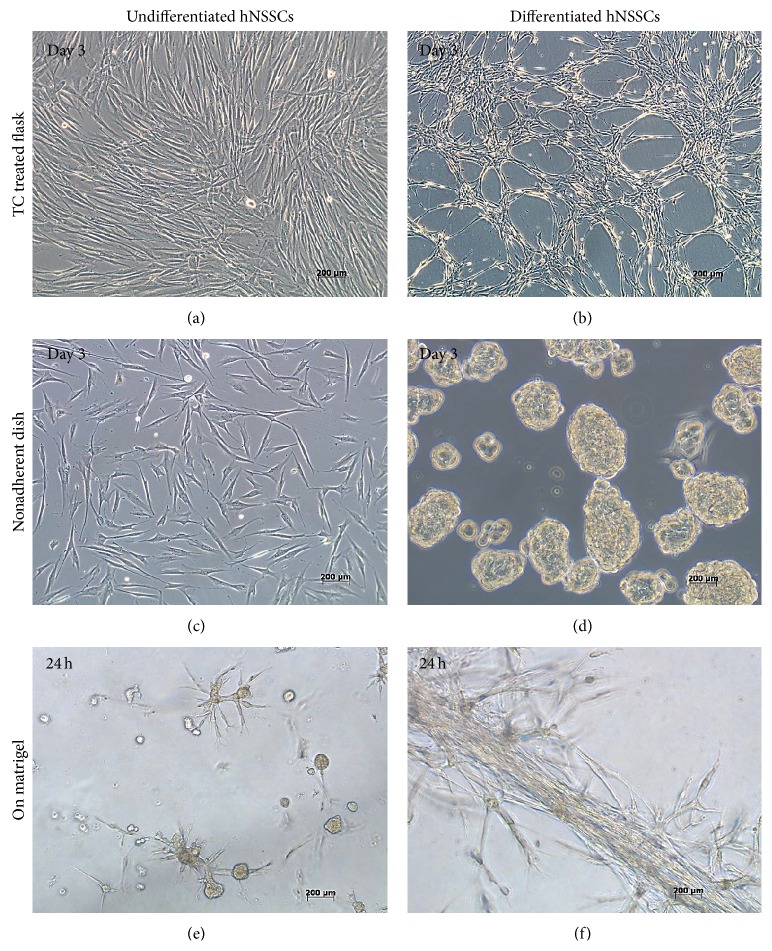
Undifferentiated and differentiated hNSSCs exhibit different morphological changes when cultured on different surfaces. Undifferentiated and differentiated hNSSCs were cultured on tissue culture-treated (a and b), or untreated (c and d) or on matrigel (e and f). Morphological changes were subsequently observed and imaged (Bar = 200 *μ*m).

Interestingly, undifferentiated and endothelial-differentiated hNSSCs displayed distinct morphology upon placement on treated and nontreated tissue culture plastic surface. Undifferentiated hNSSCs maintained their spindle shape when cultured on treated and nontreated tissue cultures surfaces (Figures 2(a) and 2(c)), while those cells exhibited modest tube formation potential when plated on matrigel (Figures 2(e)). On the other hand, endothelial-differentiated cells were observed to form matrix-rich tubular-like structure on tissue culture treated plastic surface (Figure 2(b)) and to form spheres when cultured on nontissue culture treated surface (Figure 2(d)). Most notably, differentiated hNSSCs formed tight tubular-like structure when plated on matrigel (Figure 2(f)).

### 3.3. hNSSCs Contribute to* Ex Vivo* Vasculogenesis

**Figure 3:**
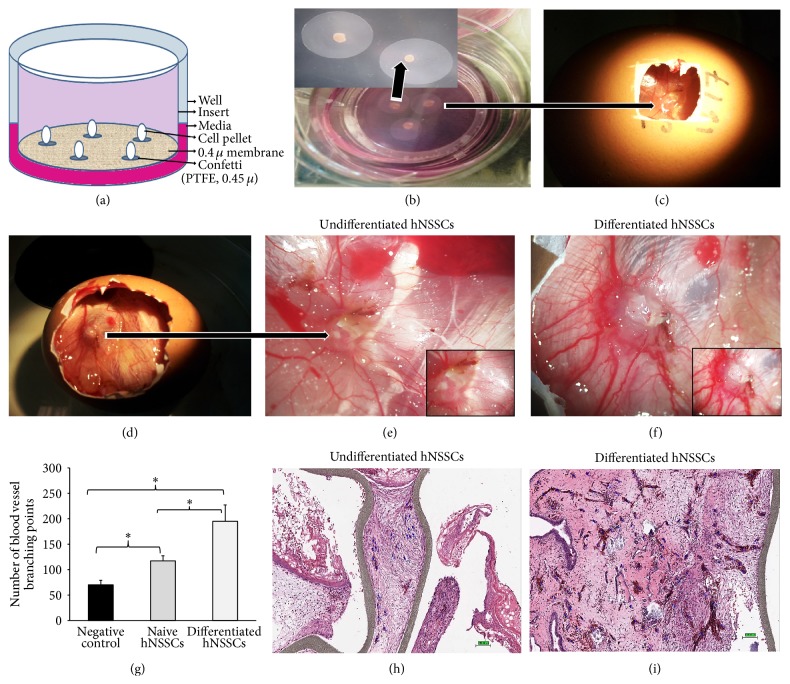
hNSSCs contribute to neovasculature using* ex vivo* CAM model. (a) Schematic illustration of the organotypic culture system (liquid-air interface). hNSSCs pellets were placed on the top of a confetti that is in contact with the membrane on the insert. The media diffuse across the insert membrane and confetti to reach the pellet. Microscopic aspect of chick chorioallantoic membrane (CAM) and organotypic cultured hNSSCs with confetti grafted to it. (b) hNSSCs pellet placed on top of a piece of confetti that is in contact with the membrane on the insert. (c) Window (2 cm^2^) made in the egg shell of a 10-day-old chick embryo with organotypic culture transplanted. (d) After 10 days, implantation shell was opened and blood vessel points were observed; (e and f) note the wheel-spoke pattern of CAM blood vessels around the graft (undifferentiated and differentiated). Stimulation of angiogenesis in CAM by undifferentiated or differentiated hNSSCs. The expansion of new blood vessels was determined by counting branch points after 10 days of cell transplantation. Sprouting and branching vessels are prominent in the CAM transplanted by differentiated hNSSCs compared to the undifferentiated hNSSCs transplantation. (g) Quantification of new blood vessels formed by naive (undifferentiated) and differentiated hNSSCs compared to negative control (confetti without cell transplantation) (*n* = 5). ^**∗**^ *P* < 0.05. (h, i) Paraffin sections of both undifferentiated and differentiated hNSSCs transplanted CAM were stained by haematoxylin and eosin stain to show the vascular density in CAM; blood vessels were counted again (blue colour points indicate vasculature) with Aperio's ImageScope software (Aperio Technologies, Vista, CA, USA) (Bar = 100 *μ*m).

**Figure 4:**
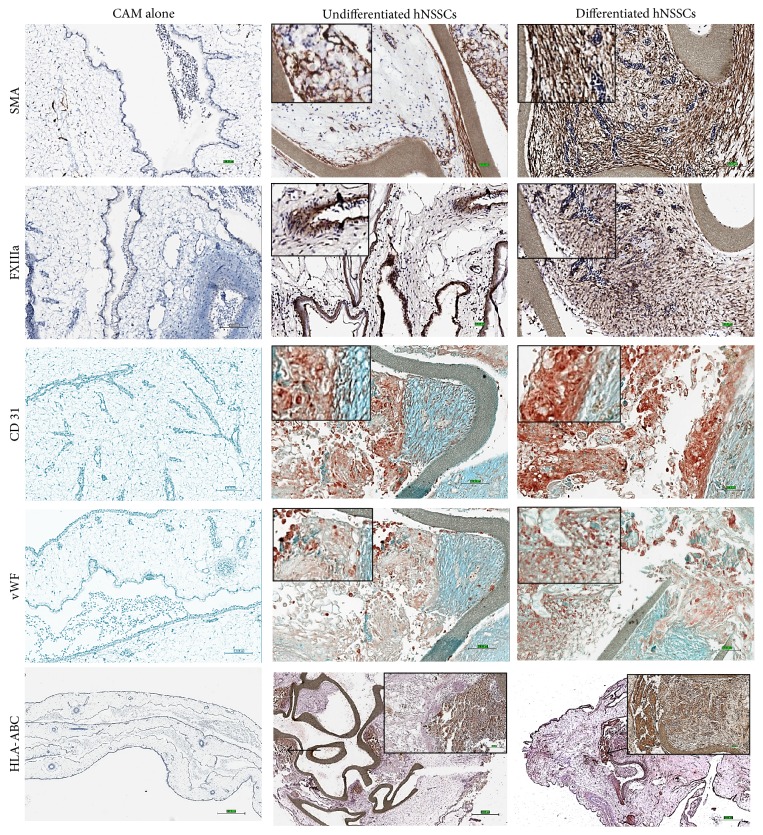
Immunohistochemical staining of CAM alone and CAM grafted with hNSSCs for endothelial-related markers. CAMs transplanted with undifferentiated and differentiated hNSSCs (10 days) were processed and stained using the indicated antibody. Brown colour indicates areas with positive staining for the respective cell marker (smooth muscle actin (SMA), factor XIIIa, CD31, and von Willebrand factor (vWF)). The human leukocyte antigen (HLA-ABC) was used to differentiate the engraftment of human cells from chick cells (Bar = 100 *μ*m).

We have previously shown the ability of hNSSCs to differentiate into endothelial-like cells* in vitro* [8]; however, the ability of hNSSCs to participate in neovasculature* ex vivo* has not been addressed to date. To assess hNSSCs* ex vivo* differentiation potential, the pellets of hNSSCs undifferentiated or differentiated under endothelial-induction conditions were cultured on PTFE confetti for 2 days as shown in schematic diagram (Figures 3(a) and 3(b)). Subsequently, cell pellets with confetti were transplanted onto CAMs by making 2 cm^2^ window on ten-day-old chick embryos (Figure 3(c)); after a further ten days of growth, CAMs were imaged and assessed for vascular formation (Figures 3(d)–3(f)). CAMs transplanted with differentiated and undifferentiated hNSSCs exhibited significant blood vessel formation. The number of vessel branching points was significantly (*P* ≤ 0.05) higher in CAMs transplanted with differentiated hNSSCs (190 ± branching points) compared to CAMs transplanted with undifferentiated hNSSCs (110 ± branching points, Figure 3(g)). Control CAM cultures displayed the lowest number of branching points (70 ± branching points). Similarly, immunohistochemical staining revealed larger number of blood vessels in CAMs transplanted with differentiated hNSSCs compared to CAMs transplanted with undifferentiated hNSSCs (Figures 3(i) and 3(h), resp.). In order to assess the contribution of hNSSCs to CAM's neovasculature, immunohistochemical investigations of CAMs were undertaken and, as shown in Figure 4, the presence of a significant number of human cells within and in regions surrounding neovessels was observed. Cells positive for the following human angiogenic markers: vWF, CD31, SMA, and FXIIIa were observed, whereas the use of the human antibody against HLA-ABC indicated the engraftment of hNSSCs in chick CAM without notable cross reactivity with the chick cells (Figure 4). Concordant with data presented in Figure 3, CAMs transplanted with differentiated cells showed higher numbers of blood vessels compared to CAMs transplanted with undifferentiated hNSSCs.

### 3.4. CAMs Transplanted with Undifferentiated hNSSCs Exhibited Epidermal Tissue Formation* Ex Vivo*

**Figure 5:**
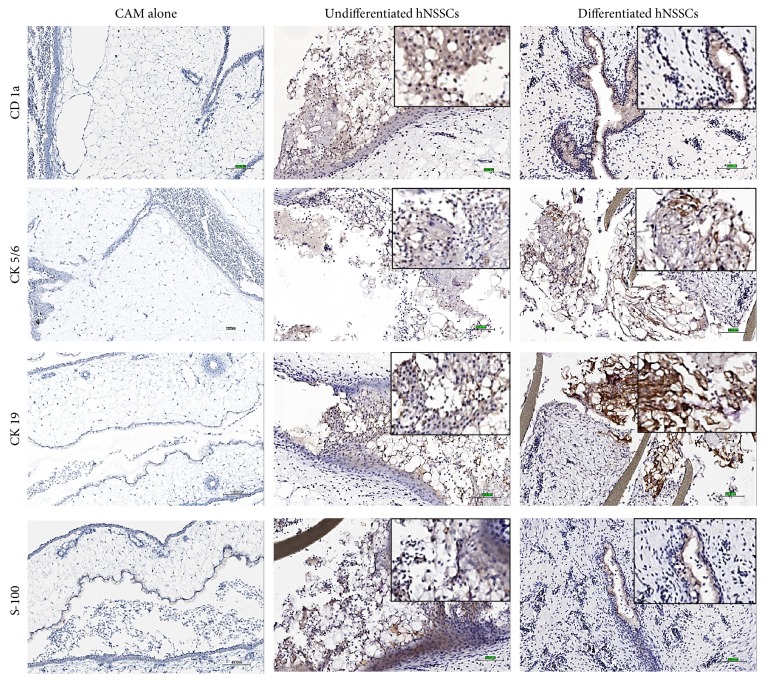
Immunohistochemical staining of CAM alone and CAM grafted hNSSCs for ectoderm-related markers. CAMs were grown as in Figure 4 and subsequently were processed for IHC. Brown colour indicates the positive area of respective skin lineage associated proteins, CD1a, CK 5/6, CK19, and S-100. CK: Cytokeratin (Bar = 100 *μ*m).

CAMs transplanted with undifferentiated hNSSCs exhibited modest staining for angiogenic markers and a modest number of neovessels (Figure 4, middle panel). Interestingly, large numbers of transplanted undifferentiated hNSSCs were observed in association with epithelial-like structures with irregular vasculature and an epidermal like phenotype that stained positively for CD1a, CK5/6, CK19, and S-100 (Figure 5, middle panel). The chick and CAM were observed to be negative for the human CD1a, CK5/6, CK19, and S-100 further suggesting that undifferentiated hNSSCs displayed epidermal potential. Studying the epidermal potential of the hNSSCs population in depth will be the subject of an independent investigation.

## 4. Discussion

Angiogenesis plays pivotal role in a number of physiological and pathological conditions. Throughout adulthood, angiogenesis takes place in response to several physiological stimuli including hypoxia and inflammation, in wound healing and in tissue regeneration [20, 21]. Therefore, enhanced understanding of the molecular mechanisms and the cell types key in the modulation and regulation of angiogenesis is pivotal in a number of areas including tumour growth and regenerative medicine.

Neonatal skin stromal cells have recently emerged as a novel source of multipotent stem cells, with the potential to form a number of distinct lineages* in vitro* [22]. Thus neonatal skin derived CD13^+^ CD73^+^ CD29^+^ CD44^+^ CD105^+^ CD90^+^ CD45^−^ CD34^−^ CD31^−^ CD14^−^ HLADR^−^ stromal cells are capable of differentiating into endothelial cells and forming CD31^+^ VE cadherin^+^ VEGF^+^ vWF^+^ eNOS^+^ capillary tube-like structures in* in vitro* [8, 22]. However, understanding the precise contribution of hNSSCs to angiogenesis under* ex vivo* conditions remains unclear. The current study shows the potential of hNSSCs to aid the angiogenic process. The ALDH assay has been widely utilized as a functional marker to isolate cancer and normal stem cells and progenitor cells. ALDH positive cells were shown to be capable of self-renewal and to possess broad lineage differentiation potential [18, 19]. In the current study, hNSSCs displayed significant numbers of ALDH^high^ cells, suggesting the presence of undifferentiated/progenitor cells. The application of an* in vitro* transwell migration and scratch assays demonstrated that hNSSCs possessed a high migration potential. The migratory potential of hNSSCs suggests additional roles for these cells in contributing to a number of repair mechanisms, including wound healing.

The current study also suggests the potential of hNSSCs to differentiate along the endothelial lineage following placement* ex vivo* in an angiogenic niche, namely, the CAM. The CAM assay has long been established as a model for the study of tumor angiogenesis and metastasis, given the absence of a fully developed immune system in the chick at this early developmental stage [11]. In addition, the CAM is a robust, cost-effective facile model to enable scaffold, factor, and cell screening, providing an intermediate approach between simple* in vitro* and complex* ex vivo* approaches [13, 23]. In this study, the presence of hNSSCs in neoangiogenesis in the CAM model was observed. Interestingly, extensive neoangiogenesis was observed with differentiated hNSSCs cultured under endothelial induction media prior to transplantation. The enhanced endothelial formation supports our earlier report of the angiogenic potential of hNSSCs* in vitro* [8]. Undifferentiated and differentiated hNSSCs were observed to augment angiogenesis in the CAM model. Cells positive for human vWF, CD31, SMA, and Factor XIIIa were observed in the endothelium of CAMs. Factor XIIIa has been shown to play a vital role in embryo implantation and to contribute to tissue remodelling and wound healing and to developmental processes that all involve angiogenesis. Factor XIIIa is regarded as a novel proangiogenic factor and has been shown to promote endothelial cell proliferation and migration and to inhibit apoptosis [24, 25]. Factor XIIIa has also been reported to be produced by multiple cell types including bone marrow derived cells and skin lineages. Factor XIIIa was observed in hypodermis of fetal skin at 6 weeks gestational ages and also in the subepidermal cellular network [26]. In the current study, we observed enhanced expression of Factor XIIIa stain in both undifferentiated and differentiated hNSSCs transplanted CAMs with increased intensity in CAMs transplanted with differentiated cells.

Differentiated hNSSCs exhibited an enhanced localization with the CAM blood vessel and were shown to exert a greater angiogenic effect than undifferentiated hNSSCs. In addition, although untested, the paracrine factors expressed by hNSSCs may also contribute to angiogenesis development, rather than direct differentiation into endothelial cells, though this hypothesis warrants further investigation.

Aside from that, undifferentiated hNSSCs exhibited epidermal differentiation capability as evidenced by CD1a, CK5/6, CK19, and S-100. Interestingly, these markers were previously linked to dermal and epidermal cells types such as melanocytes, keratinocytes, Langerhans cells, and other epithelial cells [27, 28]. However, these results require further* in vivo* validation to confirm if hNSSCs could find new application in areas such as wound healing.

## 5. Conclusion

The current study demonstrates for the first time the ability of hNSSCs to contribute to angiogenesis in an* ex vivo* system. Proliferation, differentiation, migration, cell-cell adhesion, cell-matrix interactions, and morphological regulation of endothelial cells are the important biological processes that drive vasculogenesis and angiogenesis. Our previous and current studies indicate that* in vitro* and* ex vivo* differentiation of hNSSCs offers a useful resource to explore the mechanisms underlying cell biological regulation of angiogenesis. Our previous studies have shown, at least* in vitro*, that hNSSCs are multipotent, and the current study suggests the potential of those cells to differentiate into endothelial-like cells* ex vivo*. Thus, hNSSCs are a valuable cell resource that might be useful in applications requiring enhanced angiogenesis or in areas such as ischemic diseases. Furthermore, these cells could be employed in tissue engineering and cell based therapy in which vascularization is an essential component.

## Supplementary Material

Supplementary Figure 1: Experimental negative controls for endothelial and skin lineage associated markers performed in combination with CAM immunohistochemistry. (Bar=100μm).
Supplementary Figure 2: Experimental positive controls for respective antibodies to analyze endothelial and skin lineage associated markers performed in combination with CAM immunohistochemistry. (Bar=100μm).
