# Supplementary figures and images for: Angiogenic Potential of Human Neonatal Foreskin Stromal Cells in the Chick Embryo Chorioallantoic Membrane Model
Source: Stem Cells Int. 2015 Jun 29;2015:257019. doi: 10.1155/2015/257019 (PMC4499640; doi:10.1155/2015/257019)

# Experiment Negative Controls

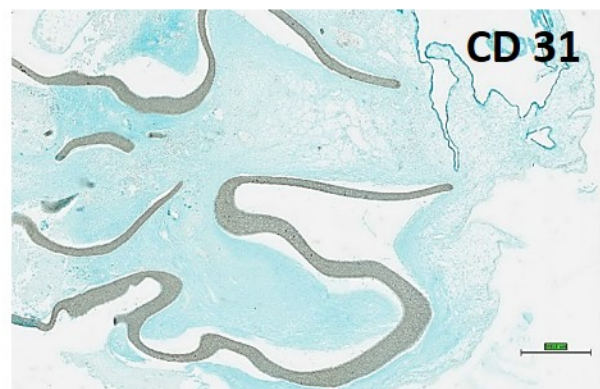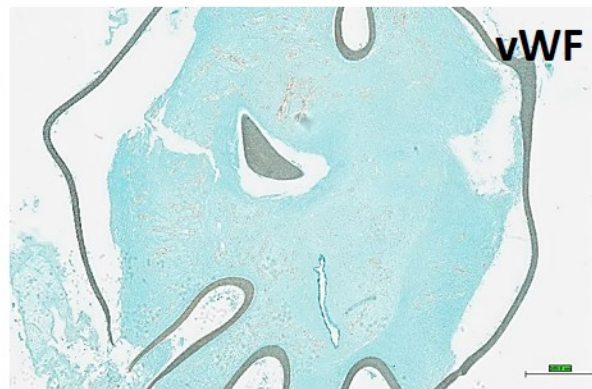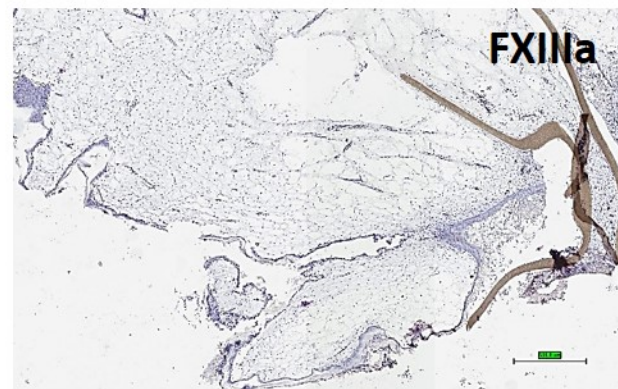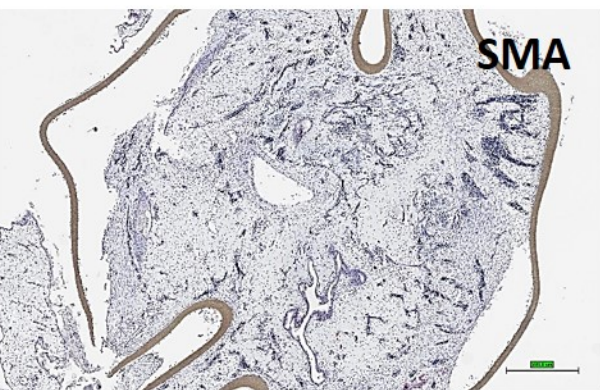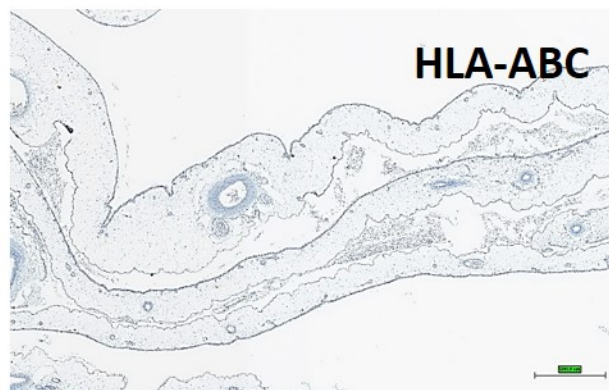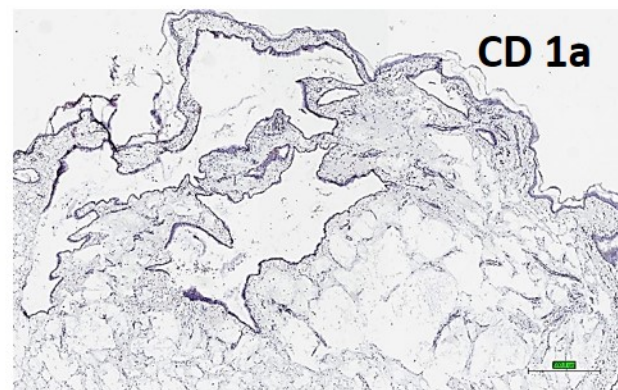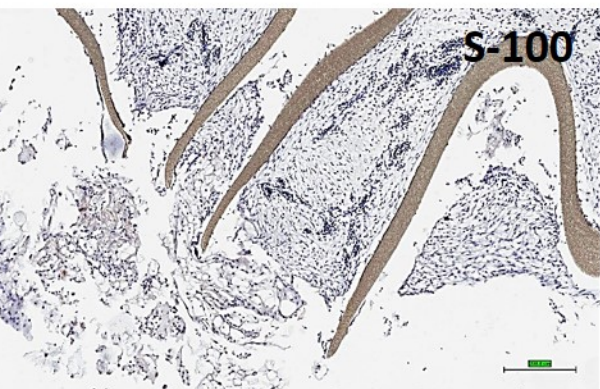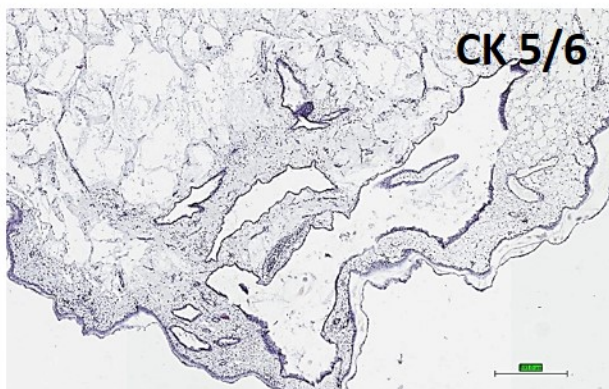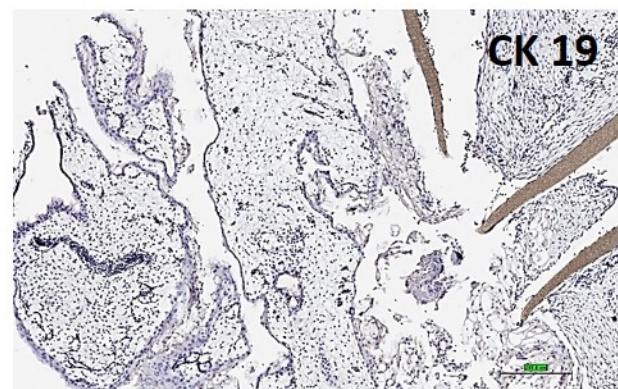

Sup Fig 1

## Experiment Positive Controls

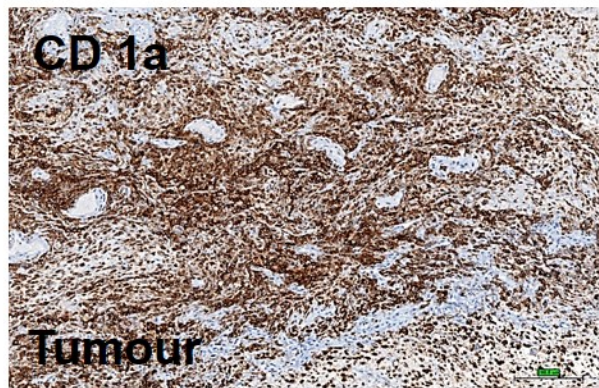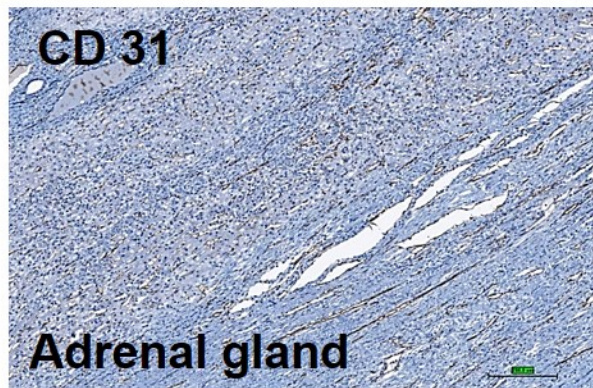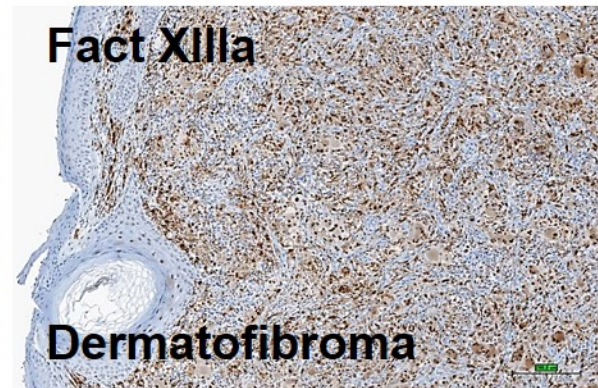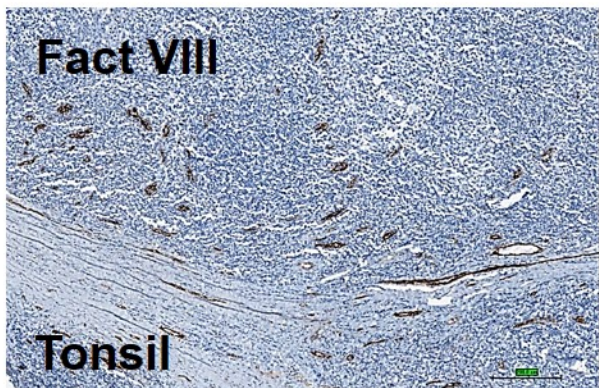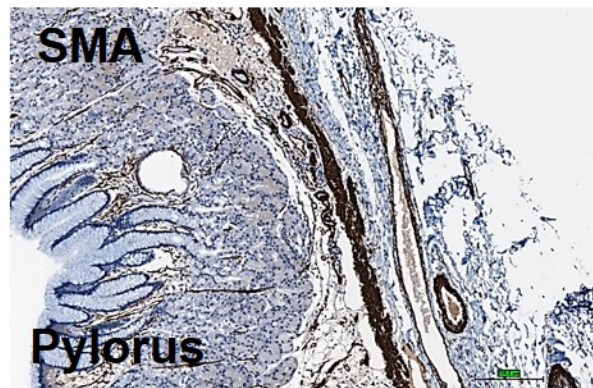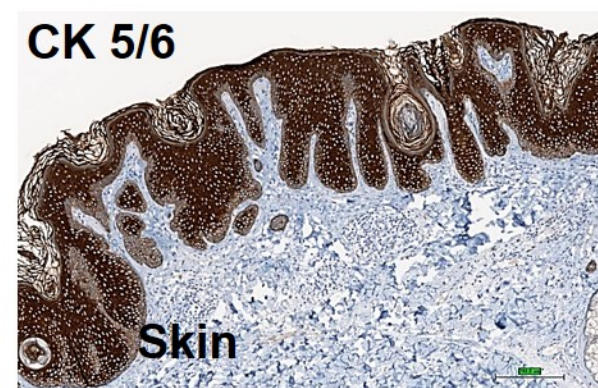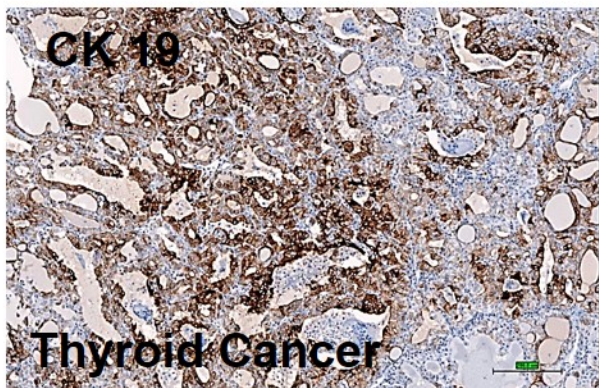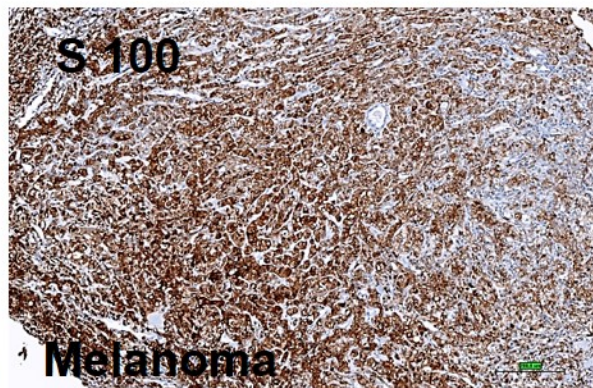

Supplement: Supplementary file 1 — Supplementary Figure 1: Experimental negative controls for endothelial and skin lineage associated markers performed in combination with CAM immunohistochemistry. (Bar=100μm). Supplementary Figure 2: Experimental positive controls for respective antibodies to analyze endothelial and skin lineage associated markers performed in combination with CAM immunohistochemistry. (Bar=100μm). [file 257019.f1.pdf]
